# Foreign Medico-Psychological Literature

**Published:** 1863-10

**Authors:** 


### FOREIGN MEDICO-PSYCHOLOGICAL LITERATURE.

Our Retrospect of current Foreign Medico-Psychological Literature embraces the following subjects:—

1. A Synoptical View of Recent Works on Epilepsy, and of J. Falret's Physiological Theory.
2. Observations on the Mental Diseases of Criminals.
3. Suicide in the United States.

1.—*Synoptical View of Recent Works on Epilepsy, and of J. Falret's Physiological Theory.*—By Prof. Albers, of Bonn.

The important works published recently regarding epilepsy are to be looked upon as the continuation of the investigations commenced by Marshall Hall, who first placed its seat in the medulla oblongata. This view seems to derive additional support from the experiments of Brown-Sequard, but in the study of this disease many well-ascertained facts of idiopathic cerebral epilepsy have been lost sight of, as well as those cases where the post mortems exhibited nothing explanatory of the epileptic condition but the degeneration of the brain and its tissues. Dr. Albers briefly gives an account of an epileptic attack, according to Marshall Hall, and, calling in question his view, points out the value of fixing decidedly its first symptoms. Is it, he asks, the cry, the paleness of the countenance, the loss of consciousness, the twitchings of the muscles of the face, or do all these occur at the same time? All authors, however, it is to be remarked, recognise the importance of loss of consciousness, which cannot be attributed to congestion of the brain, dependent upon spasm of the muscles of respiration; and, in fact, no one who has observed those cases of commencing epilepsy in which temporary giddiness and loss of consciousness occur, and in which the patient continues the interrupted step on recovering himself, can agree with Marshall Hall. We must, therefore, recognise in the loss of consciousness the far more essential and primary symptom of epilepsy, and seek another cause for it. Now this Claude Bernard thinks consequent on the want of blood in the brain and the effect of the venous stasis, which is principally injurious through the carbonization of the blood. Most writers, indeed, have adopted this view, but they differ in regard to the transition into, and the cause of, the symptoms during the second period. Thus, on the one hand, Schroeder Van der Kolk maintains there is cerebral congestion; whilst Radcliffe, Kussmaul and Tenner hold that anæmia of the brain is the cause of the convulsions. Dr. Radcliffe's view, however, is in direct contradiction to that of other observers; as, in place of holding that the muscle in a state of rest is relaxed, and the contraction consequent upon the irritating action of the nerve, he believes that the condition of the muscle left to itself is that of contraction, and that the nerve has no other action than to determine its relaxation, and that thus during the epileptic attack the medulla oblongata, instead of an over-excitement of its activity, has its energy diminished, and leaving the muscles in their natural state of contraction, is thus the cause of tonic or clonic spasms.* In other respects, however, Dr. Radcliffe adopts nearly the same explanation of the symptoms as other writers. In the first period, he assumes a want of blood in the brain, and, consequently, an excitement of its faculties from the deficiency of arterial blood; in the second, the activity depends upon venous congestion, and thus the only cause of all the symptoms of epilepsy is dependent upon the excited state of the cerebral functions, and the want of arterial blood, the natural brain irritant.

But all these opinions, however different, are supported by the

---

* Professor Albers quite mistakes Dr. Radcliffe's view of muscular action. Dr. Radcliffe holds that *living* muscle has an electromotive power, and that the electricity produced in the living muscle, *when the muscle is left to itself*, produces the state of *muscular relaxation* by keeping the molecules of the muscle in the state of mutual repulsion. He holds, also, that the muscular electricity is suspended when the muscle contracts, permanently in rigor mortis, temporarily in temporary forms of muscular contraction, and that this suspension of muscular electricity produces muscular contraction by allowing the attractive force which is inherent in the physical constitution of the muscular molecules to come into play. What Dr. Radcliffe holds is, not that the condition of *living* muscles when left to itself is that of contraction, as Dr. Albers supposes, but that this state of contraction is only possible when the *life* of the muscle is either suspended or extinguished—suspended in ordinary contraction, extinguished in rigor mortis. He does not confound life and electricity, but he shows that the vitality of the muscle and the electricity of the muscle are obedient to one and the same law; that the two ascend and descend in the strictest manner, *pari passu*. Dr. Radcliffe also holds that living nerve, like living muscle, has an inherent power of producing electricity, that this electricity, which is present during a state of inaction and absent during a state of action, may *help* the electricity of the muscle to preserve the state of muscular relaxation, and that the way in which the nerves bring about contraction is by discharging the electricity of the contracting muscle—a way which Dr. Radcliffe makes intelligible enough, but of which the limits of this footnote do not permit us to speak. Nor do these limits permit us to explain how Dr. Albers errs with respect to the statement of Dr. Radcliffe's pathological conclusions. We recommend to Dr. Albers' notice, as the most recent and satisfactory statement of Dr. Radcliffe's very important investigations, the lectures recently delivered by Dr. Radcliffe before the Royal College of Physicians in London, and published subsequently in the *Lancet*, for we gather from what is said in this article that there are many points in which Dr. Albers will sympathize with Dr. Radcliffe when they are more fully placed *en rapport*.—Ed.

same proofs, whether these are drawn from human physiology, experiments upon animals, chemical, pathological, or microscopical observations, and all these different ways of looking at the subject depend upon the new discovery which has placed the seat of epilepsy in the medulla oblongata. Observers, whilst approximating the clonic spasms of epilepsy and of other convulsive diseases, have determined that the organs which are the central points of physiological acts are also the cause of pathological acts. Indeed, pursuing this analogy still further, they do not regard the morbid irritability of the medulla oblongata as the primary cause of the morbid actions, but recognise that every reflex act includes two conditions: the impression a sensitive nerve receives, and its transmission to a motor nerve; and that to excite the sensibility of the medulla oblongata, and set it in action, it must receive an impression from a remote part of the nervous system, which, if they cannot discover in some of the nerves of the limbs or bowels, they seek in the brain.

These views are supported by experiments on the nervous and circulatory system, to the first of which belong those of Brown-Séquard, who concludes from them "that to produce spasmodic attacks in animals whose spinal cord is injured, we must irritate the terminations of the cutaneous nerves in the face, and not the larger branches. It is, besides, a most remarkable fact, that these convulsive attacks once produced may last for years, and occur spontaneously several times in a day,—every two days, every week, or every month, in the same way as we observe the return of the epileptic attack. The injured animals are thus in the same condition as individuals who have become epileptic in consequence of injury of the limbs, trunk, or head."

The experiments on the circulatory system were partly made before the seat of epilepsy was placed in the medulla oblongata, and were conducted by Sir Astley Cooper and Kussmaul and Tenner. The first of these investigators observed, that if both the vertebral arteries were compressed, after tying the carotids, convulsions took place, but disappeared when the pressure upon the arteries was diminished, from which he concluded, that want of blood in the brain is an actual cause of convulsions. Kussmaul and Tenner arrived at the same conclusions, supporting themselves principally upon the convulsions occurring in criminals during bleeding and after the opening of both carotids.

In addition to these experiments, different writers upon this subject support their views by numerous cases of epilepsy, in which a direct source of irritation is found at the periphery, and in which there was observed at the commencement of the fit an aura epileptica aut sensoria aut motoria, and not content with quoting true cases of peripheral epilepsy, they endeavour to find in much more doubtful instances the source of irritation in every organ of the abdomen and in every part of the body. Until the last three years writers confined themselves to these three kinds of proofs, when Van der Kolk sought to establish his views by the microscopical anatomy of the medulla oblongata, and from his investigations he concluded that the vessels of the nervus hypoglossus, corpus olivare, and the raphe of the nervus vagus are certainly dilated in comparison to those of the healthy spinal cord. He also believes that so long as the morbid change in the medulla oblongata consists of a simple hyperæmia, which is rather the effect than cause of epilepsy, that the disease is curable. But as every attack of epilepsy increases the hyperæmia, so every new attack diminishes the prospect of cure. By degrees also albuminous deposits form round the dilated capillary vessels, and the pathological dilatation—the consequence of the primitive hyperæmia—passes at last into fatty degeneration, or becomes indurated, and the disease is incurable.

But the question arises, is there no primary epilepsy of the brain? no cases in which a purely cerebral disease existed primarily and caused the functional disturbances of the medulla oblongata, which gave the disease in its paroxysms the form of epilepsy? This Dr. Albers does not hesitate to say there is, and quotes seven cases, four of which occurred in his own practice. From these he concludes that, as the epileptic attack began with loss of consciousness, we cannot do otherwise than assume that the disturbance originated in the brain, and that the disease was associated secondarily with disease of the medulla oblongata.

In the views of the writers previously quoted, however much they may agree in regard to the commencement of the attack, they differ in the explanation of the symptoms in the second and third stages. Brown-Séquard attributed these to the irritating effect of the carbonic acid in the venous blood acting upon the base of the brain. Van der Kolk from the increased influx of arterial blood stimulating the medulla oblongata. Dr. Radcliffe, and Kussmaul and Tenner, on the contrary, hold there is a want of blood in the second period as in the first; and Radcliffe endeavours to prove that the function of the medulla oblongata is impeded at the time there is a defect of arterial blood in the brain, and that the muscular system, deprived of the influence of the nervous system, manifests its activity in spasmodic motions. Kussmaul and Tenner, on the contrary, take the view that anæmia of the brain awakens the reflex action of the medulla oblongata, and induces a modification of its nourishment.

But these numerous differences must lead us to reflect before we adopt the opinions of those who explain the phenomena of the epileptic attack by the reflex action of the medulla oblongata, and especially so when we see how their views differ when they treat of the conditions under which this excitability arises, and when we inquire into the accidental causes necessary to transform these obscure conditions into the perfect disease. Each observer, in fact, lays too much weight upon the two elements peculiar to the reflex power of the medulla oblongata—the capability of receiving impressions and reflecting them upon the muscles—probably injured separately during the epileptic attack. Besides, there are other elements requiring explanation; the point of origin of the impression setting this excited reflex action into activity, and the modification of the circulation or of the nourishment which makes the medulla oblongata more or less capable of transferring the impressions received under the form of convulsions to the muscular system. Now all the authors quoted believe that the point of excitation of the epileptic attack may proceed from the periphery—the ganglia of the sympathetic—the spinal cord—the brain itself—or from moral causes in which mental premonitory symptoms precede the attack — indicating clearly a participation of the cerebrum. But what is now the proportion of these different causes, and is the mode of origin peripheral or central, and what is the nature and origin of the change in the medulla oblongata? According to Van der Kolk, the over-excitement of the medulla oblongata may arise from the influence of a part of a nerve, sometimes in the periphery and sometimes in the brain, and from this arises, in every epileptic attack, a marked arterial congestion in the part, which, repeated at every paroxysm, ends in permanent dilatation of the vessels; on this follows albuminous exudations through the walls of the capillaries, which either become hard, soften, or undergo fatty degeneration. These, however, are not to be looked upon as the causes of the disease, but the consequences of the primitive malady, which is first dynamic, and consists of arterial congestion, which causes a disturbance of the nourishment of the ganglion cells of the medulla oblongata. Kussmaul and Tenner, and Radcliffe, on the contrary, look upon anæmia of the brain as the cause of convulsions, and they do not agree with other observers in regard to the manner in which the symptoms of the attack are developed.

Differing, however, as these physiologists do amongst themselves, they also differ entirely from the account given by Marshall Hall of the sequence of the symptoms. The loss of consciousness precedes the attack, and those cases where this manifestation and giddiness occur, is incompatible with his theory. Indeed, from the contradictory statements made by the different authors, we are inclined to believe that the seat of epilepsy is in the brain, as we cannot conceive this paralysed in its intellectual and moral faculties, and irritated in its motor manifestations, and the difficulty is not lessened if we place the seat of the disease in the medulla oblongata. Besides, it is a peculiar assumption that the arterial congestion—venous stasis, or anæmia—which paralyses, according to the different authors, the brain, during the different periods of the epileptic attack, should irritate the medulla oblongata. To remove this antagonism, we must assume that the upper parts of the brain are anæmic, and those at the base hyperæmic; or believe with Dr. Radcliffe, that the functions of the brain and medulla oblongata are paralysed, and that the convulsive movements originate from the spontaneous activity of the muscles. Even those who agree with Brown-Séquard in his view, as M. Falret remarks, require to explain why in those cases where epilepsy exists, as a giddiness or loss of consciousness without spasm, the medulla oblongata acts alone upon the vaso-motor system, and not upon the cerebro-spinal, to which the spasms are to be referred.

All the other symptoms of the first period are equally a matter of dispute, and we find the same difference of opinion in reference to the appearances in the second. Marshall Hall attributes them to the cerebral congestion consequent on restricted breathing. Brown-Séquard assumes a venous congestion which irritates the brain, particularly at its base, and strengthens the violence of the convulsions which first originated from nervous irritation. By degrees, however, the brain is dulled by the stases of the venous blood, when the convulsions vanish and coma appears, producing the stertorous breathing, and then the toxic condition of the blood is a powerful cause of the second and third stage of epilepsy. Van der Kolk assumes both a venous and arterial congestion of the brain, and Radcliffe, Kussmaul and Tenner remain true to their doctrine of anæmia of the brain during this period. It is according to them the want of arterial blood in the brain and medulla oblongata which is the cause of all the different stages of epilepsy. But even if these views were established, are they sufficient to explain the mechanism of the attack and the origin of epilepsy? In reply we have only to turn our attention to the symptoms which follow and precede the attacks, as well as those existing in the intervals between them, to refer to the brain as the principal cause of those symptoms. The most important symptom is in reality the loss of consciousness rather than the spasm. The latter now and then occurs in imperfect epilepsy, but loss of consciousness is far more frequent as the numerous cases of epilepsia ambulatoria, gyrans, vertiginosa, saltatoria prove; and besides the post mortems of many such cases and of other epileptics have exhibited no organic change but in the brain. The observers in favour of the opposite view have principally taken into consideration those cases of sympathetic epilepsy in which the excitant to disease is in a part more or less distant from the brain, and although we admit the authenticity of such cases, we must at the same time acknowledge that all these peripheral changes and injuries occur without exciting epileptic attacks. But even when these attacks occur a definite change must exist in the brain before it is possible for the point at the periphery to produce the fit.

The recognition of the seat of epilepsy in the brain is also confirmed by the intellectual and moral condition of the patients. The frequent disturbance of their mental activity—their vehemence—their frequently eccentric moral disposition—must at once strike every observer. Falret has also pointed out the relation existing in epileptics between disturbances of the mental activity and motor power. The greater number of such patients exhibit at different times special moral and intellectual disturbances which indicate the disease as distinctly as the convulsive movements or loss of consciousness. They even alternate with these symptoms, often partly replace them, and are the outward manifestations of the same morbid condition which would prove its existence if the habitual symptoms did not exist. They all lead, however, to the same conclusion, that epilepsy is essentially a cerebral disease, in which, if modern physiology requires for the explanation of the spasmodic attacks the medulla oblongata with its reflex power, still, that this occupies only a secondary position and is subordinate to disease of the brain.—*Allgemeine Zeitschrift für Psychiatrie*, Band xix. p. 545.

2.—*Observations on the Mental Diseases of Criminals.* — By Dr. Moriz, Physician to the Board of Health and Prison at Graudenz.*

The mental diseases of criminals have many peculiarities. In many cases a connexion between insanity and crime is observable. A period of intense excitement is often accompanied by self-accusations of real or fancied crime, then by a cessation from work without reason, rebellion against the rules of the house, &c., founded upon a delusive idea of innocence and unjust condemnation, and succeeded by a persecuting and, in rarer instances, a poisoning mania directed against the jail officials and others with whom the convicts come in contact; they confuse their own identity, and often imagine themselves the victims of the crimes which they have themselves committed. These phenomena are characteristic of insanity in criminals, while hallucinations—in confinement more frequently of hearing than of vision—are common to the insane everywhere.

When fantasy stands in connexion with crime, which is generally the case when crime is the consequence of passion, an uncontrollable longing after freedom, or rather lawlessness, is a marked feature, and one which certainly betokens the commencement of insanity. It impels phantasy to action, is often mistaken by the officials for simulation, but disappears only very gradually, manifesting its presence even during stages of depression, and at last passes into incurable consecutive insanity, and paralysis of all the powers of the mind. In other cases, mental derangement developes itself as the result of unfavourable circumstances during imprisonment, and is in no way connected with the commission of bygone crime, although the afore-mentioned symptoms of insanity are not wanting.

During the last six years not a single well-authenticated case of pretended insanity has occurred in the prison at Graudenz, probably from the exaggerated idea of the difficulty in keeping up the deception, as well as the knowledge that it would not necessarily procure exemption from work. On the contrary, there were frequent instances of prisoners in the first and subsequent stages of insanity insisting upon their own soundness of mind, begging often for a shower-bath, but entreating also to be discharged from the infirmary as perfectly sane. At the same time, they complained of all sorts of bodily ailments, which existed only in their own imaginations, while the mental disease was well developed.

The silent system and the strict discipline which often prevent the detection of the earlier symptoms of insanity; the constant assumption that the eccentricities of individuals are mere simulation, the necessary segregation of such in separate cells—the only plan to use isolation as a means of cure—taken in connexion with the knowledge of guilt, form momentum sufficient to favour the development of insanity in prisons; in the same way that the sedentary life and

---

* *Einige Bemerkungen betreffend die Geisteskrankheiten Genfangenen.* Vom Kreis-Physicus, Sanitatsrath Dr. Moriz, Strafanstalts-Arzt in Graudenz.

influences inseparable from confinement induce diverse bodily diseases, which again, in many cases, form the basis of mental derangement.

In the prisons at Graudenz, which, on an average, during the last six and a-half years, contained 1200 convicts in the jail, and 150 criminals in the reformatory, there have occurred 48 cases of insanity; of these, 13 were of a mild type—*i.e.*, real but passing fits of derangement, awakening considerable doubt as to the responsibility of the individuals; and which might easily be added to the numerous cases of cure which have been recorded in other prisons. Cases also occurred frequently of inmates being attacked by what in prison slang is known by the name of the "jail crack," a state of mind recognised by the convicts themselves as a sure sign of approaching insanity. Of the remaining 33, 13 were of unsound mind at the time of their committal, and, in all probability, before the commission of their crimes; of these, 2 males and 3 females were inmates of the reformatory; 3 males and 25 women of the jail. It may be remarked, that from 1856 to 1860, among 1500 prisoners, there were about 300 females; but after that date, the proportion of females is higher—viz., about 400 in 1400. Of the 13 last mentioned, 5 were returned to their homes after a professional verdict, and 4 after the expiry of their sentence; 2 were transferred from the reformatory to the district asylum, and 2 remained in the reformatory. There were 20 cases in which insanity came on subsequent to imprisonment, of which only 2 were females. Of these 20, 5 recovered; 3, however, only partially; 5 died; 2 returned home on the expiry of their time, admission to the district lunatic asylum not being attainable; 8 remain in jail undergoing their sentence of from 1 to 9 years of imprisonment, and in the case of one young man for life.

These 20 cases may be classified as follows:—

Delirium, 5,—of whom 1 was committed for homicide, 2 for robbery, 2 for theft.

Mania, 7,—2 for murder, 2 robbery, 3 theft.

Melancholia, 8,—1 homicide, 1 attempt at murder, 1 robbery, 1 arson.

Imbecility—1 committed for theft.

In respect to age and length of residence in prison, they may be classified as follows:—From 20 to 25, 4 became insane after two years' imprisonment; 1 after 7.—From 25 to 30, 1 after 1 year; 2 after 8; 2 after 9; 1 after 13.—From 30 to 40, 2 after 2 years; 3 after 40; 1 after 6.

Lastly, 3, 50 years of age, became insane in about six months, these had often been committed for theft before.

Now comes the practical and important question.

Ought such prisoners to be in jail or in institutions for the insane?

The authorities in lunatic asylums object very strongly to the reception of such patients, fearing a prejudicial influence on the morality of their other inmates, and asserting that the means of guarding and controlling the convicts are more defective with them than in prisons, since lunatic asylums are usually only provided with attendants at the rate of 12 to 100 patients. Against the reception of those still under examination, they cannot, of course, make any objections. And, in the case of criminals, whose insanity is satisfactorily proved, it is not easy to see where their proper place is to be found, if not in an institution for the insane; since all moral responsibility must have ceased, so soon as the consciousness of guilt or punishment disappeared, a continuation of confinement in prison being, in such cases, of no possible use, and only increasing the malady. Even the infirmary of a prison is an unsuitable abode for the criminal insane, because their excitement would disturb that rest and quiet required during the progress of the bodily disease of the ordinary prisoners; and they would, in turn, suffer from the want of that attendance and the absence of those medical appointments called for by their own condition. Between two evils we must choose the least; and since it is impossible to deny that the bad habits and perverse inclinations of insane criminals are very similar to those which prevail among inmates of lunatic asylums, who have never been cited before a court of justice; and in the absence of the separate institutions for insane criminals—so urgently pleaded for by Delbrück and Damerow, in the *Allgemeine Zeitschrift für Psychiatrie*—there seems no alternative but that the district asylums for the care of the insane should be held bound to receive insane criminals, and to provide for whatever extent of separation they deem necessary between them and the other inmates. This conclusion is all the more necessary on reflecting, that after the expiry of their sentence of punishment, these convicts must, as a matter of course, under a professional verdict, be made over to the district asylums.

There is, however, a large number of cases which cannot be included among the incurable and irresponsible insane. These individuals have, through their crimes, excluded themselves from the privileges of free citizens, and from their right to the benefits of hospitals for the cure of mental disease. So long as they are aware of their situation in prison—so long has the confinement a distinct signification for them; and their degree of mental disease offers no greater hindrance to the completion of their punishment than physical disease would. We conclude, then, first—that criminals who have been pronounced, according to professional judgment, incurably insane, ought to be included with patients discharged as incurable from the hospitals for the cure of the insane, in one category; both have ceased to be men, and belong, because they once were men, to the lunatic asylum and not to the prison. To supply every house of correction, however, with suitable arrangements for the cure of the insane, similar to what is always provided for the physically sick, would, principally for want of room, be impossible, and would besides materially affect the very constitution of our penal institutions.

Although it appears in general almost impossible so to place the mentally diseased in a prison that he can be provided with the treatment and care considered indispensable to his cure, still experience teaches that, even without the full appliances to be met with in a hospital for the insane, cures are sometimes effected even in prison, in the same way that the physically diseased do recover in the jail infirmaries, although, in comparison with the extensive arrangements of richly-endowed hospitals, such infirmaries, to avoid burdening honourable citizens more than is necessary, must make shift with those arrangements only which are indispensable. In like manner must whatever is absolutely necessary for the welfare of the mentally diseased be provided in prison. There are several objections to the indiscriminate removal of all criminals suffering from different degrees of mental disease from prison, and transferring them to the district lunatic asylums, or to the newly organized asylums for insane criminals—as the possibility of such a transfer would form a powerful incentive to the simulation of insanity. There would besides be great difficulty in separating the criminal from the other patients when both required the same treatment; and again, the great increase of expense would be an almost insuperable obstacle. The prisons at Graudenz contain at present about 1100 prisoners, of these there are 9 insane and 2 of dubious responsibility. The average number during the last six years agrees tolerably well with the accounts given by Delbrück in No. 11, page 188, of the *Allgemeiner Zeitschrift für Psychiatrie*. Dr. Wichern's notes from the official reports of prisons, show that a similar computation over the whole State, with its forty prisons, would give a proportion of 198 to 242 cases of mental disease among 23,388 prisoners. The erection of a new building extensive enough to contain so many or rather double the number, as the yearly additions to the inmates would far exceed the dismissals—and as the district asylums would still try to evade receiving the criminal insane on the expiry of their sentence—would entail an almost impossible expense.

If, however, it were conceded that it is necessary only to remove the incurably insane from prison, of whom there are at Graudenz from 2 to 3, and in the whole State only from 52 to 63, it might be possible to give up one of the jails entirely to their use. It would of course be indispensable that its direction should be confided to a medical man, as it would be improper to conduct such an asylum on prison principles.

On the other hand, it is still a question, as said before, on what account the district asylums, where the accommodation so far exceeds what can be provided in prison, and where all the appliances for the humane treatment and security of the insane are already provided, should refuse to receive incurably insane criminals, and by a partition wall, or some such inexpensive arrangement, separate them from their other inmates.

As the medical treatment in a lunatic asylum consists principally—when the physical basis of disease is not evident—in the removal of all irritating influences from the patient, it must be the chief care of the medical superintendent to protect insane criminals even in prison from the like disturbing element, difficult as the task necessarily will be.

It is impossible to lay down rules for guidance in every case, as each may require a different treatment; still a few general remarks may not be out of place.

I. Where it is advisable to change the immediate surroundings of a patient, a judicious removal into a different work-ward may have a good effect, although work-wards in themselves are no fitting abode for the insane, as in spite of strict discipline and the silent system, it is quite impossible to defend the morally afflicted from the mockery of coarse companions and from the consequent excitement.

II. The infirmary, although the most suitable place for the patient so long as he requires medical treatment, is always disliked by tho insane, the sight of physical suffering producing a painful effect upon them. Besides, the enforced idleness in a sick ward is very prejudicial, creating intense weariness, and leading to continual self-inspection, to which the patient is already too prone. The insane are often eager for employment. A chintz-weaver, suffering from complete inaction of the brain, was placed in the infirmary. After a month's residence he began to eat and walk. Next he learnt to whisper a few words. Then he sang the whole day in a low voice, repeating only the words "weave chintz." When his desire was granted, his whole face lightened up; he opened wide the half-shut eyes and worked unceasingly at the weaving frame, without, however, ever performing a day's full work, or taking the slightest notice of the people around him. In the stages of convalescence, insane patients may be employed, with the best effect, in performing little household duties in the sick wards; they will often wash dishes, &c., with the most scrupulous care.

III. As solitary cells are in prisons generally on the ground-floor, and deficient in ventilation, they should be very sparingly used for confining the insane. Besides, isolation for any protracted time induces self-inspection and delusion of all kinds.

IV. Maniacs ought never to be left alone, but strictly watched in a separate apartment—less dangerous prisoners may be employed to do so, provided their compassion is excited for the unfortunate patient, whose malady, were he left solitary, would increase with great rapidity.

V. The insane should never be placed in a room where any one is seriously ill. The *Medical Journal of the Society of Physicians*, at Speyer, gives an account in No. 96, for 1861, page 765, of the beneficial results arising from the experiment of placing insane patients under the charge of families educated for the business. Some such arrangement might be attempted in prison, although of course intercourse with members of society who are at large is impossible.

In the infirmaries of all large prisons a class of patients are to be found, who, although not suffering from acute disease, are chronic invalids—afflicted with incontinentia urinæ, epilepsy, and so forth; they cannot remain in the work-wards as they are unable to comply with strict discipline, or to bear exposure to the weather, &c. It might be a beneficial arrangement to set apart a ward for their use, and to place the insane in the same room. They require similar indulgences, and it is often quite touching to observe the attention and self-denying kindness which these two classes of sufferers bestow upon each other. This would be a suitable residence for all classes of the insane except the maniac, the feeling of compulsion would be in a great measure removed, and thus bodily health could be watched over with ease and care. If it be objected that this arrangement would materially interfere with prison discipline and the silent system, let it be also remembered how greatly it would lessen the expense of the infirmary, were these two classes accommodated with a separate ward, where they could together pursue their regular work.

VI. The prison should, as a matter of course, be provided with whatever appliances are absolutely necessary for the curative treatment of the insane. A bath-room, with proper arrangements for warm, cold, shower baths, &c., in direct connexion with the infirmary, is quite indispensable.—*Casper's Vierteljahrsschrift für gerichtliche und offentliche Medicin,* Band xxii. Heft 2. October, 1862.

---

### 3.—*Suicide in the United States.*

The preliminary report of the eighth and last national census of the United States gives statistics of suicides occurring during the year ending the 31st May, 1860, which enable us to compare the phases of suicide in that country with those which are exhibited in Europe.* Although no doubt the number of suicides is considerably understated in the census returns, yet it is notorious that the moral atmosphere of America is more favourable to the crop of homicides and murders than to that of suicide. M. Majee, in his work on suicide in Bavaria, lays it down that the frequency of suicide is in inverse ratio to the frequency of crimes of violence. Where, therefore, (as in the United and Confederate States,) homicide is a common occurrence, we may expect to find a scarcity of suicides. The census records 1002 cases of suicide occurring in the year 1859-60, which gives only 3·2 to every 100,000 of the population; in England and Wales the proportion is 6·7 to every 100,000; in France the rate averages 8·3; in Prussia the rate is about 11 or 12 per 100,000; in the New England States, taken separately, (whose returns are as accurate as those of Europe,) we find the rate per 100,000 is 7·7. But we must remember this is the great "suicide field" of America: while in our London suicide field the rate is 9·7; in our midland suicide field, 8·4.*

Now as to sex. Of these 1002 suicides, 794 were committed by males, and 208 by females; that is, only a little more than a fifth of the whole were females. America has often been said to be the paradise of the female sex. These figures confirm the statement. Mr. J. N. Radcliffe states that in England and France male suicides

---

* See the articles on the "Method of Statistics of Suicide," *Journal of Psychological Medicine*, vol. xii. p. 209; the "Distribution of Suicide in England," *ibid.*, p. 470; "English Suicide Fields," *Medical Critic*, vol. ii. p. 701; and "Suicide in Bavaria," *ibid.*, vol. iii. p. 579.

are about two-thirds commoner than female; that is to say, the proportion of female suicides in the latter countries is to males as three to five: in the United States it is but little more than one to five. In the Old World a cast-off mistress commits suicide; in the New she shoots her paramour amid the applause of the community. In some States a woman can compel the father of her illegitimate child to marry her or go to prison. It is not only the law but public opinion and the juries which everywhere espouse the cause of the woman in the United States.

Has this anything to do with the small proportion of female suicides in America? Have our bastardy laws, so severe upon the woman, anything to do with the high proportion that rules among us?

Next, as to the method of suicide. Of the 1002 cases, the *modus operandi* in 299 instances (238 males and 61 females) are unspecified. The balance are thus classified:—

| | Males. | Females. | Aggregate. |
|---|---|---|---|
| Cutting throat | 57 | 10 | 67 |
| Drowning | 40 | 31 | 71 |
| Fire-arms | 109 | 4 | 113 |
| Hanging | 249 | 55 | 304 |
| Poison | 99 | 46 | 145 |
| Strangulation | 2 | 1 | 3 |

Here we find that hanging is the favourite suicidal resort with both sexes in the United States. This is also the case in every country of Europe of which we have an account, except Sardinia and Geneva, which give the palm to fire-arms. Poison, which occupies the fourth place in English and the seventh in French statistics, holds the second place in the United States. We presume that in that "free country" the laws against the indiscriminate sale of poisons are neither so universal, nor, where existing, so well carried out as in Europe. Observe how much more common, in proportion, is the resort to poison among American females than among the males. In England cutting the throat; on the Continent, generally speaking, drowning holds the second place. The fashionable poison in America is strychnine, next arsenic; in England, opium or one of its preparations, then prussic acid. In the proportion of those of both sexes who prefer mechanical means of dying, there is not much difference between England and the United States. Fire-arms, which stand second with the Saxon, and fifth with the English, stand third with the Americans, as with the French, Belgians, Danes, and many parts of Germany.

On the relation between the rate of suicide and the degree of instruction among any given community, these American returns tell an instructive tale. They entirely corroborate the dicta of M. Lisle, Mr. Radcliffe, and M. Majee, that the greater the diffusion of instruction the higher the rate of suicide. Thus, take the States where common and collegiate instruction has struck its roots most deeply, where literary tastes have been most cultivated, viz., the New England States and New York, and compare their rate of suicide with the six most populous slave-holding States. The rate of suicide in the former group is as seven to three in the latter in the ratio of their population. Thus :—

| | Population. | | Suicides. |
|---|---|---|---|
| New England and New York . . . . | 6,957,400 | ... | 370 |
| Virginia, Missouri, Kentucky, Tennessee, Georgia, and Alabama . . . . . . . | 7,065,302 | ... | 165 |

Doubtless, the returns are more accurate for the first group of States than the second, but it would be straining the point too much to contend that this alone will account for such an immense difference of rate—a difference, too, which throughout the whole list of the thirty-four States varies uniformly with the degree of civilisation each State has reached. Kentucky and Massachusetts have both well-established registrations of births, deaths, and marriages. Both States have long been habituated to reporting cases of suicide. These two States stand thus in respect to population and suicides :—

| | Population. | | Suicides. |
|---|---|---|---|
| Massachusetts . . . . . . . | 1,221,464 | ... | 110 |
| Kentucky . . . . . . . . . | 1,155,684 | ... | 32 |

The State of Kentucky has a higher rate of suicide than any other Southern State, presumably on account of the registration system which obtains there alone of the Southern States (except South Carolina), and which has habituated her people to report suicides. Thus Virginia, which had 450,000 more people than Kentucky, reports one suicide less. We may, therefore, consider the Kentucky returns as comparatively accurate. Those of Massachusetts and Rhode Island are the most accurate in the Union. Under these circumstances we find about seven suicides in Massachusetts to two in Kentucky, with an equal number of citizens.—*Social Science Review.*

